# Is phenomenal consciousness really a special case in science?

**DOI:** 10.3389/fpsyg.2024.1422050

**Published:** 2024-10-04

**Authors:** Klaus Gärtner, João L. Cordovil

**Affiliations:** ^1^Department of History and Philosophy of Science, Faculty of Sciences, University of Lisbon, Lisbon, Portugal; ^2^Center for Philosophy of Sciences at the University of Lisbon, Lisbon, Portugal

**Keywords:** phenomenal consciousness, grounding, ontological naturalism, micro-physicalism, quasi-particles, molecule structure

## Abstract

In the metaphysics of science, it is often held that higher-level properties are grounded in micro-physical properties. According to many philosophers, however, phenomenal consciousness resists this view. Many famous arguments in Philosophy of Mind have been given to reject this notion. In this paper, we argue that there is something odd about the idea that phenomenal consciousness is a special case in science and give a constructive proposal on how consciousness can fit in the natural world. To do so, we will first introduce a general notion of what grounding is. Then, we will briefly explain how the arguments for the specialness of phenomenal consciousness work by considering two famous examples, namely the zombie and the knowledge argument. In a further step, we will briefly discuss two cases from other areas in science, i.e., in particle physics and chemistry. We will demonstrate that the standard view about the reductive relation does not hold, even in these paradigm cases of the natural sciences. If what we argue is true, we think that most arguments from phenomenal consciousness cannot defeat physicalism *per se*. Finally, we will introduce an alternative way to naturalize phenomenal consciousness.

## 1 Introduction

One of the most pressing questions in Philosophy of Mind is whether phenomenal consciousness constitutes a special case in science, i.e., how to make sense of the existence of phenomenal consciousness and the idea that, ultimately, higher level properties are grounded in fundamental, micro-physical properties. It is often held that some sort of reductive *Physicalism* is the corner stone of the metaphysics of science (e.g., Berker, 2018 Carnap, 1932/33 Hempel, 1969 Papineau, 2008 Neurath, 1931 Schaffer, 2009).^1^ In our view, this is however not necessary since there are cases within science that escape this picture and phenomenal consciousness is just one very obvious case. Adopting an ontological naturalist view, we will argue that physical reductionism is not supported by our best current theories in science, and is therefore false. To do so, we will first introduce what grounding means. Then we will briefly explain the anti-physicalist line of thought by considering two potent examples, namely the zombie and the knowledge argument. In a further step, we will discuss two cases from the natural sciences in particle physics and chemistry. We will show that the standard view about the reductive relation is false. Based on this, we conclude that both, the zombie and the knowledge argument, are not good arguments against physicalism *per se*. Finally, we will introduce an alternative picture about the relation between consciousness and the physical.

In section 1, we will set up the discussion by characterizing the grounding relation and briefly introducing the zombie and the knowledge argument. In section 1.1. we will analyze the relation between higher-level properties and fundamental properties. We think that this is best done by specifying the conditions of the grounding relations entailed by micro-physicalism. In this context, we explicitly identify the following four conditions: (1) fundamentality is best described as grounding; as such (2) higher-level properties are ontologically dependent on fundamental properties; (3) higher-level properties supervene on fundamental properties; and (4) grounding is transitive and asymmetrical (Schaffer, 2012). In section 1.2., we will briefly lay out why phenomenal consciousness is often held to be problematic. We will consider two standard arguments against reductive micro-physicalism, namely the zombie argument (Chalmers, 1996, 2002) and the knowledge argument (Jackson, 1982). After a short general assessment, we will show that, in the case of our view about micro-physicalism, both arguments break with the here introduced conditions (3) and (4).

In section 2, we will consider two cases from the natural sciences. First, in section 2.1., we will analyze a case in particle physics - i.e. the case of quasi-particles. We will argue that this example constitutes an actual instance within the physical science that also breaks with conditions (3) and (4). In section 2.2., we will show that the circumstances in chemistry are similar. Consequently, we will conclude that, first, micro-physicalism is not particularly linked to the challenge of grounding consciousness (or other macro phenomena) and, second, that it fails also in the natural sciences, and is therefore false.

In section 3, we will first consider the zombie and the knowledge argument as examples of anti-physicalist arguments again and argue that they are not equipped to defeat a realistic account of physicalism. Second, we will give an alternative account of how phenomenal consciousness can be naturalized. In section 3.1, we will show that, even if consciousness does not supervene on the physical, this fact alone does not constitute a general argument against physicalism *per se*. The reason, or so we will claim, is that micro-physicalism is a bad form of physicalism and should, therefore, be considered false for the case of phenomenal consciousness as well. Finally, in section 3.2, we will paint an alternative picture. First, we will discard an obvious candidate for such an alternative, namely panpsychism (section 3.2.1). Second, we will introduce a view based on the conclusions from the case of quasi-particles and chemistry (section 3.2.2).

## 2 Physicalism, grounding and phenomenal consciousness

### 2.1 Micro-physicalism and grounding

Before we introduce why phenomenal consciousness supposedly constitutes a special case for science, we need to clarify in more detail what to be “grounded in” means. According to Schaffer (2012), a grounding relation “[...] connects the more fundamental to the less fundamental, and thereby backs a certain form of explanation” (Schaffer, 2012, p. 122). The canonical view of grounding usually assumes the relation between levels to be transitive, non-reflexive and asymmetrical, therefore allowing for a strict ordering between levels of priority. This means, if a property (A) is more fundamental than property (B) and (B) is more fundamental than (C), then (C) is grounded by (B) which itself is grounded by (A), hence, by transitivity, (C) is grounded by (A). Consequently, the notion of grounding entails a relation of priority between levels of fundamentality. According to McKenzie, fundamentality (McKenzie, 2014c; McKenzie, 2014a; McKenzie, 2014b; McKenzie, 2017) refers to a priority relation where entity (B) is ontologically dependent on or determined by a different entity (A), and (A) is neither dependent on nor determined by (B). From this follows that priority is an asymmetrical, transitive and non-reflexive relation which can take two forms, i.e., ontological dependence or determination. Further, asymmetry can be thought of from two different directions, bottom-up (e.g., atomism) and bottom-down (e.g., monism, see Schaffer, 2003; Schaffer, 2010).

Now, considering a widespread modal definition of ontological dependence, we can say the following about priority: if (B) is ontologically dependent on (A), then necessarily (B) exists only *if* (A) exists. This is of course an existential dependency where the existence of (A) is a necessary condition for the existence of (B). The existence of the fundamental level entities, therefore, is a necessary condition for the existence of the non-fundamental entities. This concept of fundamentality is specified by Schaffer, which explicitly claims that “[...] the entities of the fundamental level are *primarily* real” (Schaffer, 2003, p. 498).

In turn, if priority is spelled out as a determination relation, the idea is as follows: (B) is determined by (A) if (A) is a sufficient condition for the existence of (B). This means, to explain (B), we only need to explain (A). So to say, we get the explanation of (B) for free by explaining it in terms of (A). Therefore, settling the fundamental suffices as a condition to settle the non-fundamental. This is the paradigm case of supervenience, where properties of the most fundamental level determine properties of higher levels. Since supervenience is transitive, properties of higher levels are fully determined by properties of the fundamental level.

For a better understanding of supervenience, consider, for instance, the following case: the chemical level is grounded in the physical level in the sense that the physical level is more fundamental than the chemical. This means: (1) fundamentality, in the case of chemistry, is best understood as grounding; (2) chemical entities are ontologically dependent on micro-physical entities; (3) chemical properties supervene on micro-physical properties; and (4) grounding is a transitive,^2^ non-reflexive (i.e., chemical properties cannot ground themself) and asymmetrical (i.e., chemical properties do not ground physical properties) relation. In section 2 we will discuss micro-physicalism in more detail. There, we will maintain that all facts are grounded in microphysical facts, if they are ontologically dependent and supervenient on microphysical facts. Our goal is to show that even if we accept this standard formulation, micro-physicalism is false.

### 2.2 Phenomenal consciousness

For now, we take what was said in the last section to be the standard view about how to naturalize any higher-level property. Usually, the problem of consciousness refers to the problem of how to ground the phenomenal.^3^ In this context, phenomenal consciousness or phenomenal properties should be thought of as ‘what it is like for someone’ (Nagel, 1974) to undergo a conscious experience. To be grounded in the micro-physical means that the relation between phenomenal properties and micro-physical properties has to fulfill the above introduced four conditions for grounding, namely: (1) fundamentality, in the case of phenomenal properties, is best understood as grounding; (2) the phenomenal has to ontologically depend on the micro-physical; (3) phenomenal properties have to be determined by – or supervene on – micro-physical properties; and (4) grounding has to be transitive and asymmetrical.

To show why the case of phenomenal consciousness is supposedly special in science, we consider two famous arguments against physicalism, namely the zombie argument (Chalmers, 1996, 2002) and the knowledge argument (Jackson, 1982).^4^ *Prima facie* this anti-physicalist reasoning is designed to cast doubt about whether one can maintain that phenomenal consciousness can be grounded in the micro-physical.

In the rest of this section, we briefly set up how both arguments relating to the problem of grounding phenomenal consciousness. Consider first the zombie argument. This argument is basically based on the idea that we can imagine a world where phenomenal zombies exist. This world is a perfect copy of our world, and the zombies are perfect copies of us – therefore they behave/act the same way we do and utter the same things. However, those zombies lack phenomenal consciousness. This means, for those zombies there is nothing it is like to see, for instance, red. To refute physicalism, this argument introduces one more idea, namely that the following scenario cannot be ruled out *a priori*: since this zombie world is conceivable, it is also metaphysical possible. The easiest way to put the structure of the argument is the following:

1. Zombies are conceivable.
2. What is conceivable is possible.
3. Therefore zombies are possible (Kirk, 2023).

This argument is supposed to show that we cannot *a priori* rule out that there is a world with exact physical copies of us which, however, lacks phenomenal consciousness, i.e., making them phenomenal zombies. As a result, we cannot say that phenomenal consciousness is grounded in the micro-physical.

The second argument considered here is Franck Jackson’s knowledge argument. This argument states that even if we have all physical information about colors, when we experience them for the first time, we learn something new. Therefore not all information is physical. Here is Jackson’s famous quote about Mary the color scientist:

“Mary is a brilliant scientist who is, for whatever reason, forced to investigate the world from a black and white room via a black and white television monitor. She specializes in the neurophysiology of vision and acquires, let us suppose, all the physical information there is to obtain about what goes on when we see ripe tomatoes, or the sky, and use terms like ‘red’, ‘blue’, and so on. She discovers, for example, just which wave-length combinations from the sky stimulate the retina, and exactly how this produces via the central nervous system the contraction of the vocal chords and expulsion of air from the lungs that results in the uttering of the sentence ‘The sky is blue’. (It can hardly be denied that it is in principle possible to obtain all this physical information from black and white television, otherwise the Open University would of necessity need to use color television.) What will happen when Mary is released from her black and white room or is given a color television monitor? Will she learn anything or not? It seems just obvious that she will learn something about the world and our visual experience of it. But then it is inescapable that her previous knowledge was incomplete. But she had all the physical information. Ergo there is more to have than that, and Physicalism is false.” (Jackson, 1982, p. 130).

The general structure may therefore be put the following way:

1. Before her release, Mary, the color scientist, acquires all physical information about color vision.
2. When released, Mary learns something new about color vision, namely about visual experience.
3. Therefore: there is information that is not physical and physicalism is false.^5^

This thought experiment claims that physical truths do not entail, at least all, experiential truths. As a result, no matter how much one learns about the physical aspects of colors, it does not entail ‘what it is like for someone’ to actually perceive, for instance, red. Consequently, the physical realm does not *a priori* include the phenomenal.

This is the standard and intuitive way of understanding these arguments against physicalism. The question now is what part of the physicalist story is doubted specifically. At least in the context of the here discussed micro-physicalism, we are in the position to analyze the arguments within the context of the grounding conditions stated in the last section. In a nutshell, we have laid out that the zombie argument argues for the possibility of zombie worlds (and consequently denies physicalism) and the knowledge argument claims that physical truths do not entail all experiential truths. In our view, this means that both arguments deny conditions (3) and (4) of micro-physicalism. According to the zombie and the knowledge argument supervenience between micro-physics and phenomenal consciousness does not hold and, as a consequence, transitivity breaks down.

In the case of the knowledge argument, this is more obvious. By claiming that physical truths do not entail all experiential truths, especially implies that condition (3) is violated. Consequently, this argument leaves conditions (1) and (2) in tact. This means, first, one can be agnostic about the claim that fundamentality is best understood in terms of grounding as suggested by (1). As we will see in the next section, the important questions are whether or not we can spell out what the fundamental is and whether or not the grounding relation holds. As for (2), phenomenal properties might still depend, for their existence, on physical properties. There is no problem in assuming that experiencing, for instance, phenomenal redness depends ontologically on the physical properties of red and the perceiving person, i.e., properties such as wave length and/or brain states. It is simply not the case that the latter properties exhaust the former. This is also the reason why condition (3) fails. What follows from the knowledge argument is that the phenomenal cannot be fully determined by the micro-physical. Since without criterion (3) transitivity and asymmetry are interrupted, condition (4) collapses as well.

In the case of the zombie argument things are similar, but perhaps harder to see. Here, the claim that a zombie world is possible also undermines conditions (3) and (4). Just as in the case of the knowledge argument, there is no problem with the assumption that physical properties are ontologically prior to phenomenal properties. In both, our world and the zombie world, there are the same physical properties. The difference is that in our world phenomenal properties are instantiated and in the zombie world they are not. The instantiation, respectively non-instantiation, of phenomenal properties is the reason why condition (3) fails. If it were true that the micro-physical fully determines the phenomenal, i.e., in the end there is nothing over and above the micro-physical, then a zombie world would not be conceivable in the first place. Of course, (4) collapses as a consequence again.

In the remainder of the article, we will argue that this is less problematic than one would expect. We will show that phenomenal consciousness is not the only case in science where conditions (3) and (4) break down. Consequently, we believe that micro-physicalism is a bad version of physicalism to begin with. This means, we need alternative ways to think about physicalism in science.

## 3 Micro-physicalism in science

Consider the conditions for micro-physicalism from section 1.1. again. The conditions imply that all higher properties – i.e. phenomenal properties, chemical properties and even higher-level physical properties – are ontologically dependent and determined by the fundamental properties of micro-physics. Despite being a very intuitive thesis, this view is far from obvious. To say that a property is grounded in fundamental physics suggests (a) there is a fundamental, well determined, lower ontological level of physical reality; and (b) that there are no cases of ontological emergence.^6^ But having a closer look at science shows that there may be some indicators that this is far from the truth. This does not only concern the relation between the physical and biological or chemical level or between the physical and the conscious level, but also the different levels within physics itself. To show this, let us first consider the case of quasi-particles.

### 3.1 Micro-physicalism and the case of quasi-particles

Quasi-particles are excitations in solids. To be more precise, they are the result of a collective excitations of the subatomic constituents of a solid (in an order of 10^23^), e.g., electrons. Therefore, quasi-particles do not exist independently of solids, in particular their subatomic constituents. In this sense, “not existing independently” means that quasi-particles exist only *if* solids exist. For this reason, quasi-particles are ontologically dependent on the solid’s constituents. This means, they are not independent entities, as “standard” particles, such as electrons, photons, etc., are supposed to be. For this reason, “quasi-particles” are sometimes called “fake” entities.

However, sometimes things are not as straightforward as they seem. According to Brigitte Falkenburg, for instance, “[...] [quasi-particles] are ontologically on a par with free electrons, protons, neutrons, as well as the subatomic matter constituents of atomic, nuclear, and particle physics.” (Falkenburg, 2015, p. 248). Moreover, Falkenburg even believes that “[…] if quasi-particles are fake entities, then so are all kinds of subatomic particles, too.”

The important idea to keep in mind is that if quasi-particles are not real particles, then we need to give some criterion to distinguish them from real particles. But, can we do so straightforwardly? To answer this question we have to ask ourselves, what it means to be a “particle.” Interestingly, physics subscribes to several particle concepts (Falkenburg, 2007, 2015). The most relevant in what concerns our case are the Quantum Concept, the Operational Concept and the Symmetry Concept. Following Falkenburg, those concepts may be defined in the following manner (Falkenburg, 2015, p. 230):

Quantum Concept (QC): particles have non-local states but may be approximately localized by means of a position measurement. They propagate like waves and they are indistinguishable (Fermi/Bose statistics). Their dynamic properties are mass, momentum/energy and charge; in addition, spin and parity.

Operational Concept (OC): particles are local events in a measuring device for position measurement. Their properties are what is measured by a particle detector, namely: mass, momentum/energy and charge; in addition, spin and parity.

Symmetry Concept (SC): particles are the (irreducible) representations of symmetry groups. Their dynamic properties are the parameters that correspond to the representations: mass (or energy, respectively), spin, parity and flavor.

Of course, there are differences between these particle concepts. Consider for instance propagation. SC only refers to “free” particles, so it is not concerned with interaction between particles. OC is only committed to what is “observable” and, therefore, does not engage in inferred features such as the particle’s propagation (e.g., that particles behave like waves and hence they are indistinguishable). QC allows that particles have interactions, non-local states and that they propagate as waves.

Despite all the differences in existing particle concepts relevant to Modern Physics, these concepts are compatible. This can be seen by the following commonality: all particle concepts attribute the same properties to **all** particles. In our particular case, this leads to the question of whether or not quasi-particles have the same features. As already stated, quasi-particles are excitations in solids. Nevertheless, their properties consist of effective momentum/energy, mass, charge and spin. Moreover, quasi-particles propagate like waves and are indistinguishable. Also, they can be localized in certain conditions. Therefore, if we accept that a particle is what has the properties that are transversal to SC, OC and QC, then quasi-particles are particles like any other kind of particle. The quasi only refers to the fact that they cannot exist independently of solids.

It may, however, be objected that this is a question about the nature of quasi-particle, i.e., whether quasi-particles are real or just theoretical, mathematical entities. One example of the latter are so called virtual particles and material particles are a case of the former. But, where do quasi-particles stand? As quasi-particles, virtual particles do not exist on their own: they are relative to collective effects. But that is almost the only feature both have in common. Virtual particles are not localized; virtual particles do not obey to energy conservation; virtual particles are not measurable. Conversely, quasi-particles are localizable, obey energy conservation and parity rules and can be measured just as material particles. So, quasi-particles relate closely to material particles, i.e., real particles, and not to virtual particles.

As Falkenburg shows, in what concerns the reality of quasi-particles in experimentation, they pass Hacking’s reality criterion: “If you can spray them, they exist” (Falkenburg, 2015, p. 227). This means that, in terms of scientific experimentation, quasi-particles are just as “real” as any other quantum particle. As a matter of fact, since, on the one hand, the ontological status of quantum objects remains unclear (and the long-standing problems of realism of entities), the question “are they real?” can be put forward for all subatomic particles. On the other hand, if we consider particles as a bundle of properties, then we have already shown that quasi-particles are on the same ontological level, i.e., they are as “real” as subatomic entities.

In the light of what was said in these last paragraphs, it becomes difficult to support the standard and “intuitive” view that quasi-particles are not as “real” as real particles. One can argue that “intuition” *per se* is not an acceptable argument in philosophy, even more so when one deals with quantum entities. We still understand that, in the case of the standard view, the only way to overcome this problem is to argue that quasi-particles are not in the same way “real” as quantum particles, since quasi-particles ontologically depend – for their existence - on solids and their properties. But arguing that quasi-particles are not “real” - or at least not on the same ontological level of “real” quantum particles - rather means that quasi-particles are context-dependent, since they “emerge” from collective excitations. However, we think that the lesson to be learned here, is not so much that quasi-particles are not real, rather that they are context-dependent, i.e., they depend on collective excitations and cannot exist as independent entities. But, the same can be said about all kinds of particles. For instance, quarks, by nature, cannot exist independently (Cordovil, 2015), but they are nevertheless considered real elementary particles. Even worse is the fact that electrons, photons, muons and other kinds of particles cannot really exist in an absolute independent fashion. There are always fields, interactions and processes present and, if one endorses ontic structural realism^7^ (Esfeld and Lam, 2011; French, 2014; Ladyman and Ross, 2007), then it is no surprise that all physical particles are context-dependent since no intrinsic properties exist. So, we follow the bolt statement by Falkenburg (2015): if you think that quasi-particles are not real, then you should think the same about subatomic particles.

In the light of these considerations, the essential issue boils down to the following question: Are quasi-particles ontologically reducible to electrons? According to what we have argued in this section so far, this seems highly unlikely. We subscribe to the view that phonons (quasi-particles) and electrons are ontologically on par. As argued above, the simple reason is that both entities share the same set of properties such as spin, mass, charge, etc. If quasi-particles were reducible to electrons, we should expect electrons to have at least one property that quasi-particles lack. Since this is not the case, there is no reason to assume that there is an asymmetrical dependency relation between quasi-particles and electrons.^8^ This means, unless we can point to a specific property that electrons possess and quasi-particles lack, we cannot say that the latter supervenes on – or is fully determined by – the former. To establish supervenience, we need a more fundamental property that serves as the supervenience base. Since all the properties of quasi-particles and subatomic particles are the same under any particle concept, there is no property that can fulfill this condition.

There may be two concerns here. First, since for quasi-particles to exist, they depend on the existence of the constituents of solids, one may argue that there is nevertheless a fundamental asymmetry and therefore a property electrons have and quasi-particles lack. This means, it seems reasonable to claim that quasi-particles supervene on electrons. And second, if quasi-particles and electrons have the same properties, then emergence is off the table since there is no new, causally autonomous property. This challenge is even stronger, if we consider that the dependence between electrons and quasi-particles could go both ways. In what follows, we will show that both arguments fail.

To respond to the first claim, we suggest that to establish this well ordered existence dependency is not as simple as one may expect. As we already insinuated, it is possible that the dependence between electrons and quasi-particles could go both ways. The standard theory about quasi-particles defends that they are ontologically dependent on electrons, but as Guay and Sartenaer (2016a) argue, it may well be that electrons are composed of quasi-particles. If the existence dependency can go both ways, then – even if we concede that quasi-particles exist only when subatomic particles exist to the micro-physicalist – this does not establish an ontological asymmetry, but a form of dualism. This is rather a practical concession to the standard picture without impact on the ontological status of quasi-particles. Therefore, quasi-particles and electrons are on the same ontological level and quasi-particles are not reducible to electrons.

The second concern can be disregarded for our argumentation here. Our claim is not that quasi-particles emerge from electrons, but just that quasi-particles do not supervene nor are reducible to electrons. This has straightforward consequences. If quasi-particles are “ontologically on a par” with quantum particles then they are not reducible to them. This notion, however, is not compatible with the assumption of a fundamental level of properties (micro-level), upon which higher-level properties supervene or are grounded in. This does not violate the idea of an existential dependency, i.e., quasi-particles need the constituents of solids to exist. However, the determination/supervenience relation does not hold, i.e., quasi-particles are not fully determined by the constituents of solids.

In sum, we think it is difficult to establish that quasi-particles supervene on subatomic entities. Quasi-particles are therefore not grounded in subatomic entities and micro-physicalism or micro-reductionism is false. This is the case even in fundamental physics. Transitively, it makes no sense to defend that chemical, biological or consciousness properties are reducible to micro-physical entities and/or their properties.

### 3.2 The case of chemistry

Since the case of quasi-particles may not be the most obvious one, we want to strengthen our view by considering a more apparent case that can be found in chemistry. As Hendry (2019, 2024) noted, molecular structures are pivotal to chemistry: Chemical reactions must involve changes to molecular structures, and the chemical behavior of a compound substance is essentially a matter of understanding how its structure transforms into the structure of a different substance.

Any molecule is composed of atomic nuclei and electrons, and depends on them. Since nuclei and electrons are both quantum objects, molecular structure could be expected to be fully derivable from Quantum Mechanics (QM). Thus, it should be suspected that molecular structures are reducible to its parts: electrons and nuclei. According to Hendry (2019, 2024), however, this is not the case. As a matter of fact, bridging the relation between QM and molecular structures depends on assumptions about physical interactions entailed by electrons and nuclei within a molecule itself which are incompatible with QM. Consequently, Hendry argues that molecular structures are a case of strong ontological emergence in science. But, what is the argument?

QM describes many-body systems in terms of the Schrödinger equation. The system of electrons and nuclei is one of those cases. Therefore, a reducionist should expect that we can derive the specific structure of any molecule from the Schrödinger equation since molecules cannot be anything more than a system of electrons and nuclei. However, that is arguably not the case.

First, QM explains molecular structures using approximations, like the ‘Born-Oppenheimer’ approximation (BO-A) and the adiabatic approximation (A-A). In these approximations, electronic and nuclear motions in molecular systems are separated from how they respond to external interactions. The A-A relates to how a molecule’s electronic and nuclear motions interact. It assumes that the movement of atomic nuclei is slow compared to the movement of electrons, allowing the electrons to instantaneously adjust to the positions of the nuclei as they move. Within this approximation, the energy of a molecule changes in terms of the function of positions of its nuclei, implying that the electrons are always in their lowest energy state for that configuration. The Born-Oppenheimer approximation, on the other hand, focuses on decoupling electronic and nuclear motions to simplify the quantum mechanical description of molecules. More specifically, BO-A assumes that the nuclei are fixed in space, that is, well localized. In both approximations, there is a significant change in the system’s physics that goes beyond the scope of QM. In fact, the molecular system is ultimately semi-classic and, therefore, the molecular structure is not (and cannot be) derived from QM nor is it reducible to just the combination of electrons and nuclei under QM descriptions. As Hendry noted: “Firstly isomers, which are molecules in which the same types of atoms are bonded together in different ways, share their molecular Schrödinger equations, the starting point of the earlier explanation. Thus, ethanol CH_3_CH_2_OH and dimethyl ether (CH_3_OCH_3_) share the same Schrödinger equations, as do enantiomers such as L- and D-tartaric acid. The starting point of the explanation, the molecular Schrödinger equation, does not respect the differences between isomers” (Hendry, 2019, p. 347). That means, using only QM would not make it possible to differentiate between isomers. However, isomers are not only different entities but can also have very different causal powers. We can address isomers by considering that nuclei are localized in each molecule. However, by doing so, we change the physical situation since we change the system’s symmetry to classic symmetry, i.e., BO-A is not a case of reduction because it changes the symmetry properties of molecular structure. The Born-Oppenheimer approximation cannot be just a mere approximation since it influences the scope of the quantum-mechanical description. In this case, we consider that interaction with the rest of the system effectively transforms the nuclei from quantum entities into classical objects. Or, as Hendry puts it:

“Molecular structures are ontologically dependent on electrons and nuclei: they cannot exist without them. The novelty consists in the distinct dynamical behavior displayed by electrons and nuclei in the context of structured systems: adiabatic separability, nuclear localization and restriction to a single classical structure, which in each case is a suspension of the normal behavior of a quantum system. The adiabatic separability and localization are also examples of transformational emergence [...], in which the behavior of the parts of an emergent system is so different that it makes sense to say that they have been transformed into a new kind of entity. The radical transformation of nuclei from entities that obey quantum statistics into localized, semi-classical entities would seem to be a good example of transformational emergence” (Hendry, 2024, p. 158).

From the above, it becomes clear that molecular structures - chemical entities - are ontologically dependent on quantum entities like nuclei and electrons but are not reducible to them. Much to the contrary, molecular structures seem to ontologically emerge from micro-physical entities.

## 4 This looks like good news for conscious research

### 4.1 Forget micro-physicalism and grounding consciousness

What does the case of quasi-particles and chemistry mean for our efforts to naturalize phenomenal consciousness? Since micro-physicalism already fails in the realm of the natural sciences, it is in our opinion not surprising that it fails in general. For instance, both, the zombie argument and the knowledge argument, deny that phenomenal consciousness supervenes on the micro-physical. It seems, however, that this fact alone does not constitute a general strain of arguments against physicalism *per se*. As we have seen, quasi-particles and molecule structures do not supervene on micro-physics either. As a consequence, it may well be the case that we have to take phenomenal consciousness very seriously, i.e., the phenomenal does, in a sense, really constitute a set of properties that does not supervene on the physical.

This, however, does not mean that physicalism is wrong. Both the zombie and the knowledge argument only show that phenomenal consciousness is a further case in which supervenience fails. At this point, the reader may ask whether or not the cases from the natural sciences and phenomenal consciousness are really that similar. Therefore, we should explicitly state what the differences between both cases are and why we still think that our conclusion holds. In this context, we think that this issue boils down to the following question: Are the cases of quasi-particles, molecule structure and phenomenal consciousness comparable? On the one hand, we have anti-physicalist arguments from the realm of consciousness, which are highly based on our intuitions. On the other hand, we have laid out arguments from the natural sciences, which rely on disproving the following assumptions of micro-physicalism: (a) there is a fundamental, well determined, lower level of physical reality; and (b) that there are no cases of ontological emergence.^9^ It is true that there is a difference between these two kinds of cases. For instance, it is difficult to decide whether or not a world without quasi-particles and molecule structures is conceivable and hence possible.^10^ However, that is not the point. The similarity holds for the grounding conditions. What the zombie argument, the knowledge argument and the cases from the natural sciences have in common is that the determination/supervenience condition (3) and the transitivity/asymmetry condition (4) fail. The fact that the explanations for this issue are different, stems in our opinion from the fact that the arguments belong to different realms. We will pick up on this thought in the next section, but first let us state that we think that if we take the combination of conclusions from the cases in natural science and the here presented anti-physicalist arguments seriously, then we are left with the consequence that phenomenal properties are compatible with some version physicalism. The reason is that physicalism is not identical to micro-physicalism. This means that phenomenal consciousness does not need to be compatible with micro-physicalism. To naturalize the phenomenal, we need to deny micro-physicalism and tell a different story.

### 4.2 An alternative physicalist picture

But how would this story look like? We think there are many options. We will focus on the following two here: (i) phenomenal consciousness is just like quasi-particles and molecule structures; and (ii) phenomenal consciousness has its own story. To do so, let us first introduce a non-micro-physicalist picture that stems from what we have said in the last section. This picture is based on the idea that according to physicalism everything ontologically depends on physical properties. However - and different to micro-reductionism or micro-physicalism - everything is not determined by the properties of fundamental physics. What the case of quasi-particles and molecule structure shows is that micro physicalism is dubious even within the fundamental natural sciences and should therefore be rejected. However, neither the case of quasi-particles, nor the here portrayed case from chemistry denies ontological dependence. As a consequence, we should assume that higher level properties are also not reducible to properties of fundamental physics, but nevertheless they ontologically depend on them. Since there is an ontological dependence relation, one could claim that there are basic physical properties from which higher-level non-physical properties emerge. This means higher-level properties ontologically depend on physical properties but are not determined by them. We have argued that quasi-particles are not reducible to subatomic particles and molecule structures are neither. We claimed that the latter case may commit us to an actual case of ontological emergence. Since this last view may be considered controversial, we will not pursue this possibility here any further. An alternative interpretation is that there is only one basic physical substance on which all properties depend (physical and non-physical properties equally), but do not derive from. Hence, quasi-particles, chemical and biological properties depend on physical properties (micro-physical properties in particular), but are not reducible, nor determined by them. Consciousness is should not be considered to be any different.

As stated in the previous section, the zombie argument and the knowledge argument against supervenience may attack micro-physicalism from a different angle, but according to what we have argued in the context of the natural sciences their conclusion should be expected. The question is whether the case of phenomenal consciousness is analogous to the case of quasi-particles and molecule structures or whether it must be handled in its own right. We will show that there are some important insights that we can take away from the idea of an analogy, but they have to be incorporated into a narrative specific to consciousness.^11^

#### 4.2.1 Is consciousness “on par” with micro-physics?

So, let us consider whether or not the case of consciousness is like the case of quasi-particles and molecule structures (i). Here, we are examining in a sense the fundamental level of physics. The question is if we can describe a strict hierarchy of levels of composition. The case of quasi-particles has shown that this is not as easy as one intuitively thinks. As we have argued, quasi-particles are not composed of other particles, i.e., phonons are not constituted by electrons. Further, we stated that molecule structures cannot be reduced to QM descriptions. But does this affect our view about grounding phenomenal consciousness? To answer this question let us start with a fundamental difference between the case of quasi-particles, molecule structures and consciousness. As we have claimed in section 2.2., it is difficult to ground quasi-particles in “real” particles, or better there is no good criteria for doing so. Quasi-particles fulfill all the conditions of “real” particles, and this is the case under any given particle concept. Furthermore, ontological dependence may go either way. The only thing we can say is that supervenience fails. Also, we argued that we cannot allow for a supervenience explanation for molecule structures in terms of QM.

Consciousness obviously does not have straightforwardly the same properties as physical or chemical entities. This means, there are distinguishing criteria. But, does this mean that we have a clear cut way to describe what is more fundamental? We think not. One reason is that according to both the zombie argument and the knowledge argument supervenience also does not hold in the case of consciousness. Since supervenience does not hold the question could arise – even if we do not necessarily think so – whether or not phenomenal properties and physical properties are on par.^12^ In this context, consider for instance panpsychism. Usually this view is discarded as a form of physicalism,^13^ since it claims that phenomenal properties are just as fundamental as physical properties and cannot be reduced to them. This is an idea that, at least, micro-physicalism cannot accept. Furthermore, panpsychism states that phenomenal properties can be found all over the place in nature just like physical properties. These two statements are not only problematic for many physicalists, it makes panpsychism also substantially different to dualism. While dualism advocates for the fundamental difference between mind and matter, panpsychism assumes a unified picture of nature, including the phenomenal and the physical (Goff et al., 2017). In our context, the important claim is that panpsychism assumes that phenomenal properties do not supervene on physical properties, they are both considered fundamental. This idea mirrors the case of quasi-particles. The phenomenal and micro-physical are ontologically on par.

There are, however, a few things to consider. Panpsychism always comes with the trade off that we do not know how more complex types of consciousness arise from simpler types, i.e., it is difficult to understand how my consciousness arises from the combination of the conscious properties of my parts such as cells. This is the so called combination problem (Seager, 1995). Even though this is considered one of the toughest problems to solve for panpsychism, we would like to take a more physical perspective here. The problem we see is that if we allow phenomenal consciousness to be on par with micro-physics, we probably have to allow this for other cases as well. One case that quickly comes to mind is “life.” We can see no reason why we should allow consciousness to be on par with micro-physics and life not. Our worry, however, is the following: if we allow too many properties to be on par with micro-physics, physicalism becomes trivial. It is not clear what has to count as fundamental, so technically we can fill in what we want. Some may say that consciousness and life are good examples that could exist alongside with micro-physics. But this opens the door to pack everything we do not understand into the fundamental level. Sooner or later we will wind up with a trivial ontology where nothing needs to be explained, since it is all fundamental. On our view, this is not desirable and we will therefore not defend that phenomenal consciousness is on par with micro-physics. However, we do believe that we can learn something from the case of quasi-particles and chemistry that can be incorporated in the consciousness narrative.

#### 4.2.2 The story of consciousness

Since we believe that phenomenal conscious properties do not supervene on micro-physical properties, let us try to combine what was said about quasi-particle, molecule structures and a story of consciousness in its own right (ii). We think the way to go is to incorporate two conclusions into the consciousness narrative. This means, we would like to allow for properties that are non-reducible and deny the necessity for fundamentality of levels of composition.

Let us start with non-reducibility. In section 3.2. we have already stated that if we take the cases of quasi-particles and molecule structures seriously, then we wind up with a version of physicalism where higher-level properties cannot be reduced to micro-physical properties. Even if those higher-level properties depend ontologically on the physical. This can also be applied to consciousness. One idea we want to add here is that we do not think that we have to get rid of levels in general, it is just that strictly hierarchical ordering fails.

This brings us to the idea of fundamentality. So far, we have argued in this section that phenomenal consciousness should not be regarded as being on par with micro-physics. However, within the realm of phenomenal consciousness there is a debate about what makes a mental state conscious in the first place. Chalmers’s zombie argument and Jackson’s knowledge argument assume that what is essential to consciousness are qualitative properties or “what it is like” to have an experience. So, when Mary leaves her black and white room and finally sees the blue sky, she learns what it is like to have an experience of blueness.

This, however, is not the only way of thinking about what makes a mental state conscious. Here is what Gallagher and Zahavi have to say:

experiences are characterized by a quality of *mineness* or *for-me-ness*, the fact that it is *I* who am having these experiences. All the experiences are given (at least tacitly) as *my* experiences, as experiences *I* am undergoing or living through. All of this suggests that first-person experience presents me with an immediate and non-observational access to myself, and that (phenomenal) consciousness consequently entails a (minimal) form of self-consciousness. In short, unless a mental process is pre-reflectively self-conscious there will be nothing it is like to undergo the process, and it therefore cannot be a phenomenally conscious process (Gallagher and Zahavi, 2015, p. 1).

What is claimed in this passage is that without pre-reflective self-consciousness there is no phenomenal consciousness or what it is like to undergo an experience. Consequently, what makes a mental state conscious is the fact that it possesses minimal self-consciousness (Gallagher and Zahavi, 2015; Clowes and Gärtner, 2018; Gärtner, 2018; Gärtner, 2023; Kriegel, 2009; Parnas and Sass, 2011; Sass and Parnas, 2003; Zahavi, 1999, 2005; Zahavi, 2014; Zahavi and Kriegel, 2015, Kriegel, 2005; Kriegel, 2012). In this view, it is due to the so called subjective properties of experience that consciousness arises and not phenomenal qualities like blueness.

As the reader can see there are, at least, two ways of how to think about what brings about phenomenal conscious. When we consider Nagel’s original idea that consciousness means that what it is like for someone to undergo an experience, we can either concentrate on the ‘what it is like’ or ‘for someone’ part. But, if we have learned something from the case of quasi-particles, this is not necessary. We can reasonably think that subjective properties and qualitative properties are ontologically on par.^14^ According to Nagel, phenomenal consciousness consists of both properties by definition, i.e., both have their part to play. Now, if, on the one hand, we take Gallagher’s and Zahavi’s account seriously, then subjective properties are prior to qualitative properties. To put it differently, qualitative properties need subjective properties to exist in the first place. This view, however, does not claim that the former properties are determined by the latter. On the other hand, if Chalmers and Jackson are correct, then subjective properties are nothing more than a special case of qualitative properties, namely a qualitative property that is shared by all conscious experiences, never changing and self-referential.^15^ The consequence is that subjective properties only exist because qualitative properties exist. This does not mean that qualitative properties determine subjective properties.

The question now is which of the two portrayed options describes or explains the nature of consciousness adequately. If we can learn something from the case of quasi-particles, then, or so we think, a decision between both theories is not necessary, i.e., subjective and qualitative properties may be ontologically on a par in the sense that a mental state needs both dimensions to count as conscious.^16^ To show this, consider what the above theories about the nature of consciousness argue. If portrayed correctly, both accounts only argue for the idea that either qualitative or subjective properties come first. This means they argue for an ontological dependence. However, neither of those views argues for determination, i.e., neither argues that a supposedly less fundamental property is fully determined by the supposedly more fundamental property. We have learned from the case of quasi-particle and molecule structures that dependence relation for existence is not enough to establish fundamentality. Properties are context dependent and therefore need other properties for their existence. Since both theories about the nature of consciousness can acknowledge the existence of subjective and qualitative properties, we think that neither account needs to establish that one property is more fundamental than the other. What is established are two different emphasis on Nagel’s original characterization of phenomenal properties. In our opinion, this is equivalent of describing vibrations in solids either in terms of electrons or in terms of phonons. We acknowledge that there are some basic differences between what the physical and the phenomenal is - for instance, the former entails descriptions about the fundamental structure of the world, while the latter is concerned with the nature of subjective experiences. However, if we consider a minimal continuity to be true, we think that the case of quasi-particles has shown that to think about consciousness in the context of determination is false. As a consequence, we think that an adequate theory about consciousness must take the whole Nagelian statement into account. It is not enough to concentrate on one part.

The last question still open is how these thoughts about the constitution of conscious experience help us to reconcile physicalism and the Science of Consciousness? The main idea here is that a strict ontological determination relation is not necessary, not in particle physics, not in the study of consciousness and not in chemistry. If what we have said so far is correct, then it is far from clear how so called higher-level properties can be grounded in fundamental properties, i.e., there seems to be no strict ordering of levels and different levels can be ontologically on par – even in the case of particle physics. Something similar seems to hold for the case consciousness, where different properties of the phenomenal seem to escape strict ontological determination as well. Further, if we take seriously what we have said about grounding molecular structures in QM – i.e. this idea is highly contentious – grounding consciousness in QM, or better micro-physicalism, seems also unrealistic. Our conclusion from all this is not that there is something strange going on when considering the nature of consciousness. We rather think that there are many cases in science – even in fundamental physics and other natural sciences – which point to a rejection of micro-physicalism. In our view, this means (a) micro-physicalism is false; and (b) we need to concentrate on spelling out physicalism differently. Especially (b) may even ask us to rethink the relations between different levels in science – i.e. physical, chemical, biological levels and so forth (including the conscious level) – without giving up the idea of physicalism *per se*.^17^

## 5 Conclusion

In this paper, we have argued that consciousness and physicalism can be compatible based on the fact that micro-physicalism is a bad form of physicalism, even in the realm of science. We have shown this for the cases of particle-physics and molecule structures. Finally, we have adapted our conclusions to fit the realm of consciousness and argued that there is hope for the Science of Consciousness.

## Data Availability

The original contributions presented in the study are included in the article/supplementary material, further inquiries can be directed to the corresponding author.

## Footnotes

^1^For a profound discussion of Physicalism see especially (Stoljar, 2010; Stoljar, 2024).

^2^According to Schaffer (2012), it is not clear if grounding is a transitive relation. As many other authors do (Clark and Liggins, 2012; Tahko, 2013), we assume that this is the case.

^3^This often referred to as the ‘hard problem of consciousness’ (Chalmers, 1995).

^4^Of course, this list of anti-physicalist arguments is not exhaustive.

^5^For a related structural analysis see Nida-Rümelin and Conaill (2024).

^6^For a detailed discussion of emergence see, e.g., Santos (2015, 2020, 2024).

^7^Subscribing to this view means the following: (1) one should be ontologically committed primarily to relations – even if objects are admitted, individual substances and, therefore, intrinsic properties should be dismissed; (2) one should only be a realist about the relational structures of our “best scientific theories.”

^8^A similar conclusion, based on different reasoning, is defend by Guay and Sartenaer (2016a, 2016b).

^9^We will leave aside for the moment that we have also introduced some intuitions for the case of quasi-particles.

^10^We are not even sure how to translate the knowledge argument to accommodate these cases.

^11^In our view, this may be exactly the type of reasoning that leads to the introduction of the Meta-Problem of Consciousness (Chalmers, 2018). The Meta-Problem of Consciousness asks in its most simplest version why we think that there is a ‘Hard Problem of Consciousness’ to begin with. As we will see, we opt for a different solution.

^12^Theories of consciousness based on this idea include Russellian Monism (Russell, 1914a,b), panpsychism (Brüntrup and Jaskolla, 2017) and protopanpsychism (Chalmers, 1996).

^13^This does not mean, panpsychism is necessarily discarded as a form of naturalism.

^14^Gärtner (2018) defends a view along these lines in terms of conscious experience.

^15^This view is defended in detail by Nida-Rümelin (2014).

^16^Especially, Metzinger (2020) has denied this idea. In this view, pure consciousness does not need, what he calls a minimal sense of selfhood (MPS) or pre-reflective self-consciousness to count as conscious. We think that this is an extreme view. Most philosophers seem to think that both, subjective and qualitative, aspects of consciousness must be accounted for somehow. Therefore, we will assume that if the Nagelian definition that it must be ‘What it is like for the organism’ to count as conscious experience is to be taken seriously, then we should account for both properties in some way.

^17^To give a detailed story in the case of consciousness is beyond the scope of this paper. But one of us has argued elsewhere for such an account (Gärtner, 2023).
